# Endothelial dysfunction of resistance vessels in female apolipoprotein E-deficient mice

**DOI:** 10.1186/1476-511X-9-51

**Published:** 2010-05-19

**Authors:** Maine S Cola, Agata L Gava, Silvana S Meyrelles, Elisardo C Vasquez

**Affiliations:** 1 Laboratory of Transgenes and Cardiovascular Control, Physiological Sciences Graduate Program, Health Sciences Center, Federal University of Espirito Santo, Vitoria, ES, Brazil; 2 Laboratory of Cardiovascular Pathophysiologies, Research Center, Emescam College of Health Sciences, Vitoria, ES, Brazil

## Abstract

**Background:**

The effects of hypercholesterolemia on vasomotricity in apolipoprotein E-deficient (ApoE) mice, a murine model of spontaneous atherosclerosis, are still unclear. The studies were mostly performed in conductance vessels from male mice fed a high-fat diet. In the present study, we evaluated the endothelial function of resistance vessels from normal C57BL/6 (C57) and hypercholesterolemic (ApoE) female mice in both normal and ovariectomized conditions.

**Methods:**

Twenty week-old C57 and ApoE mice underwent ovariectomy or sham surgery and were studied 30 days later. The vascular reactivities to norepinephrine (NE, 10^-9 ^to 2 × 10^-3 ^mol/L), acetylcholine (ACh) and sodium nitroprusside (SNP) (10^-10 ^to 10^-3 ^mol/L) were evaluated in the isolated mesenteric arteriolar bed through dose-response curves.

**Results:**

ACh-induced relaxation was significantly reduced (P < 0.05) in ApoE compared with C57 animals, as indicated by both the maximal response (37 ± 4% vs. 72 ± 1%) and the LogEC_50 _(-5.67 ± 0.18 vs. -6.23 ± 0.09 mol/L). Ovariectomy caused a significant impairment in ACh-induced relaxation in the C57 group (maximal response: 61 ± 4%) but did not worsen the deficient state of relaxation in ApoE animals (maximal response: 39 ± 5%). SNP-induced vasorelaxation and NE-induced vasoconstriction were similar in ApoE and C57 female mice.

**Conclusion:**

These data show an impairment of endothelial function in the resistance vessels of spontaneously atherosclerotic (ApoE-deficient) female mice compared with normal (C57) female mice. The endothelial dysfunction in hypercholesterolemic animals was so marked that ovariectomy, which impaired endothelial function in C57 mice, did not cause additional vascular damage in ApoE-deficient mice.

## Background

Atherosclerosis is a slow, progressive and multifactorial disease that results from interactions between genetic and environmental factors [1]. The murine model that lacks the gene encoding apolipoprotein E (ApoE) and spontaneously develops hypercholesterolemia and atherosclerotic lesions similar to those found in human beings [2,3] has greatly contributed to the understanding of this disease. In this animal, on a chow diet, as early as 10 weeks of age, monocyte adhesions are observed, followed by intermediate lesions containing foam and smooth muscle cells at 15 weeks and sequentially by fibrous plaques at 20 weeks of age [4]. Although endothelial dysfunction has been considered one of the early steps in atherosclerosis [5], its occurrence in ApoE-deficient mice is still controversial. Studies in conductance vessels of male and female ApoE-deficient mice have shown both normal endothelial function [6,7] and endothelial dysfunction [8-11]. On the other hand, studies in resistance vessels from ApoE-deficient mice have shown normal endothelial function in males [12,13] and endothelial dysfunction in females [14]. It is well established that estrogens exert several direct effects on the vessel wall [15-18] and that post-menopausal women [19] are known to present an elevated risk of cardiovascular events, which can be reduced by exogenous administration of 17β-estradiol [20,21]. In accordance with this view, it has been shown that the loss of female hormones leads to the impairment of endothelial function in rats [22-24], but this was not yet investigated in mice.

The importance of studying resistance vessels is based on the fact that arterioles act as control conduits through which blood is released into tissue microcirculation. Considering that the responsiveness of resistance vessels is still poorly understood in the mouse and that there are few studies in atherosclerotic female mice, the present study was designed to evaluate endothelial function in the isolated mesenteric arteriolar bed of C57BL/6 and ApoE-deficient mice under both normal and ovariectomy conditions.

## Methods

The experiments were performed using 20-24-week-old female C57BL/6 (C57) and ApoE mice obtained from the animal facilities of the Health Sciences Center at the Federal University of Espirito Santo. The animals were housed according to institutional guidelines for animal research and fed a normal diet. The procedures were previously approved by the institutional Ethics Committee for Use of Animals (CEUA-EMESCAM, protocol #002/2009) and were conducted in accordance with the international guidelines for care and use of laboratory animals.

At 20 weeks of age, C57 and ApoE mice underwent ovariectomy, as previously described [22]. Thirty days after ovariectomy, the animals were anesthetized (ketamine/xylazine, 91.0/9.1 mg/kg, *i.p.*) and blood samples were collected for determination of 17β-estradiol and total cholesterol levels. 17β-estradiol levels were determined using chemiluminescence assays (Marcos Daniel Laboratory, Vitoria, Brazil) and total cholesterol levels were measured colorimetrically using a commercial kit (Bioclin, Sao Paulo, Brazil). After blood sample collection, the superior mesenteric artery was cannulated. The mesenteric arteriolar bed was then transferred to a 37°C water container and perfused at a constant rate (2 mL/min) with oxygenated (95% O_2 _- 5% CO_2 _mixture) physiological salt solution (in mmol/L: 130 NaCl, 4.7 KCl, 1.6 CaCl_2_·2H_2_O, 1.18 KH_2_PO_4_, 4.7 MgSO_4_·7H_2_O, 14.9 NaHCO_3_, 0.026 EDTA and 11.1 glucose) using a peristaltic pump (Harvard Apparatus, Holliston, MA, USA). Perfusion pressure was monitored via a T-tube inserted between the pump and the inflow cannula and connected to a pressure transducer and a data acquisition system (BioPac Systems, Goleta, CA, USA). The dose-response curves to norepinephrine (NE, Sigma Chemical Co. Saint Luis, MO, USA, 10^-9 ^to 2 × 10^-3 ^mol/L), acetylcholine (ACh, Sigma, 10^-10 ^to 10^-3 ^mol/L) and sodium nitroprusside (SNP, Sigma, 10^-10 ^to 10^-3 ^mol/L) were then performed in the isolated mesenteric arteriolar bed. The vascular responses were evaluated as changes in the perfusion pressure and the vasodilator responses to ACh and SNP were calculated as percentages of the contractions induced by NE (5 × 10^-6 ^mol/L).

All data are expressed as means ± SEM. Statistical analysis was performed with 1- or 2-way ANOVA followed by Tukey's *post hoc *test for multiple comparisons. The level of significance was set at P < 0.05.

## Results

Body weight, uterus weight, uterus weight/body weight ratio, plasma 17β-estradiol and cholesterol levels are summarized in Table 1. Thirty days after ovariectomy, body weight was not altered; however, uterus weight was significantly reduced in ovariectomized groups, with no differences between C57 and ApoE animals. Consequently, the uterus weight/body weight ratio was diminished in about 80% in the ovariectomized groups when compared with the respective sham groups. The success of ovariectomy was also confirmed by plasma 17β-estradiol levels, which were significantly lower in ovariectomized groups (2-fold, P < 0.01) in comparison with sham animals, also without differences between C57 and ApoE mice. As expected, plasma cholesterol levels were significantly higher (6.5-fold) in ApoE than in C57 mice. Hypercholesterolemia levels of ApoE mice were not affected by ovariectomy.

**Table 1:** Body and uterus weight and plasma 17β-estradiol and cholesterol levels in C57 and ApoE-deficient mice groups

Parameters	C57		ApoE	
	
	Sham	OVX	Sham	OVX
Body weight (g)	25.9 ± 0.5 (22)	26.8 ± 0.6 (22)	26.3 ± 0.6 (12)	25.5 ± 0.7 (12)
Uterus weight (mg)	30.3 ± 1.8 (22)	5.4 ± 0.7* (22)	36.2 ± 1.6 (12)	5.9 ± 1.3^† ^(12)
Uterus weight/body weight (mg/g)	1.18 ± 0.10 (22)	0.20 ± 0.02* (22)	1.38 ± 0.07 (12)	0.23 ± 0.05^† ^(12)
Plasma 17β-estradiol (pmol/L)	128 ± 8 (14)	61 ± 4* (13)	152 ± 12 (11)	71 ± 3^† ^(9)
Plasma cholesterol (mmol/L)	1.66 ± 0.13 (16)	1.42 ± 0.11 (13)	10.77 ± 1.02* (11)	9.64 ± 0.88* (9)

Data are reported as means ± SEM of the number of animals indicated in parentheses.

OVX: ovariectomized. *P < 0.05 *vs *C57 Sham; ^†^P < 0.05 *vs *ApoE Sham (ANOVA).

**Figure 1:**
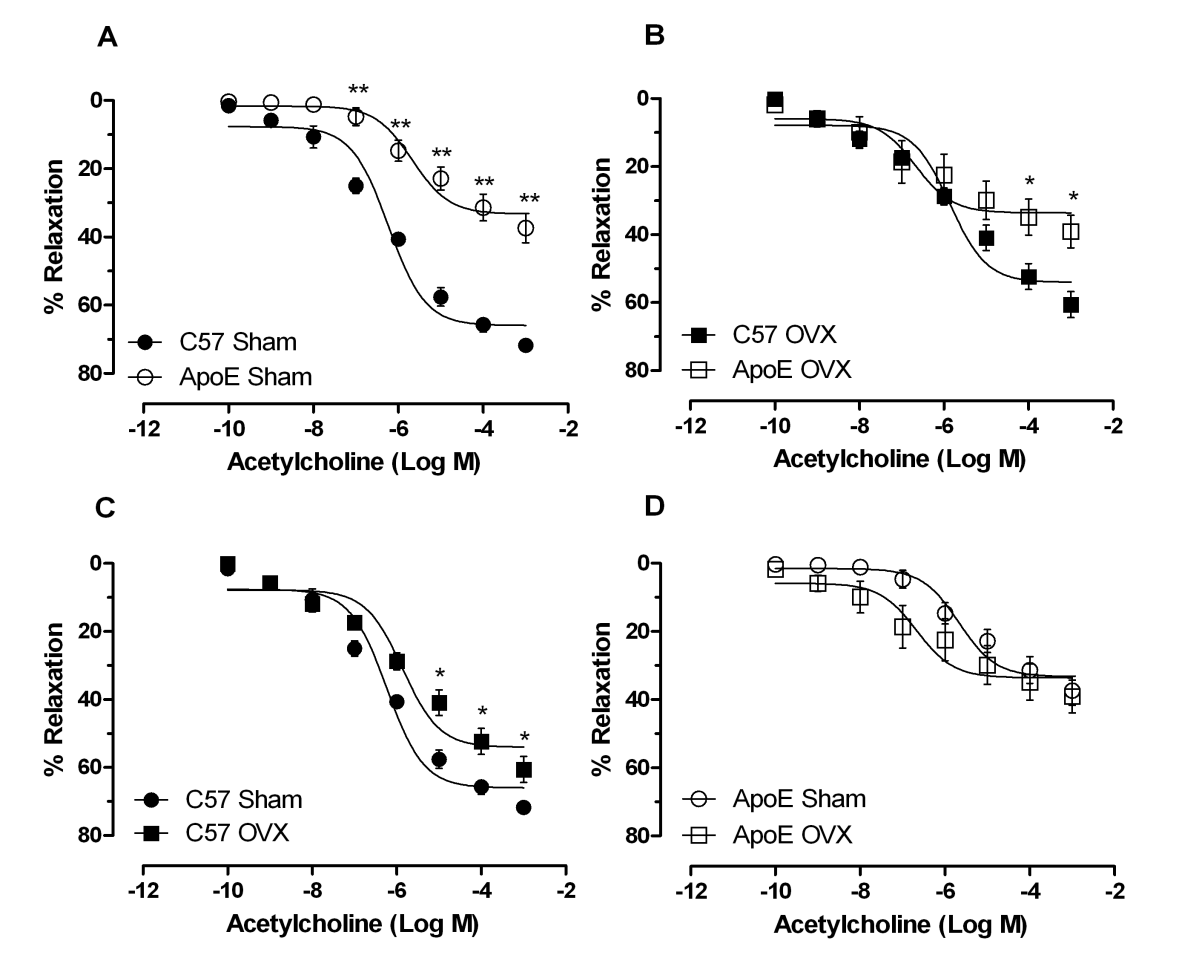
**Endothelium-dependent relaxations produced by acetylcholine in mesenteric arteries of C57 and ApoE mice 30 days after ovariectomy (OVX) or sham surgery (Sham)**. Responses are expressed as the percentage of relaxations relative to the NE-induced precontractions. A: comparison between C57 Sham and ApoE Sham. B: comparison between C57 OVX and ApoE OVX. C and D: effects of ovariectomy on acetylcholine-induced vasorelaxation in C57 and ApoE mice, respectively. Data are reported as means ± SEM (n = 8 to 10 per group). *P < 0.05, **P < 0.01 *vs *respective control (ANOVA).

The endothelium-dependent vasodilatation of the isolated mesenteric arteriolar bed in response to ACh is shown in Figure 1. The vascular responsiveness to ACh was significantly reduced in ApoE animals compared with C57 animals, as indicated by both the maximal response (37 ± 4% vs. 72 ± 1%, P < 0.01) and the LogEC_50 _(-5.67 ± 0.18 vs. -6.23 ± 0.09 mol/L, P < 0.05) (panel A). Ovariectomy caused a significant impairment in the ACh-induced relaxation in the C57 group (panel C) but did not worsen the deficient state of relaxation in ApoE animals (panel D). Even after ovariectomy, ACh-induced relaxation still was significantly diminished in ApoE animals compared with C57 animals (maximal response: 39 ± 5% vs. 61 ± 4%, P < 0.01, panel B).

The endothelium-independent vascular smooth muscle relaxation in ApoE mice produced by the nitric oxide donor SNP was statistically similar the relaxation in C57 mice in both intact and ovariectomized animals. The vascular constriction response to NE was also not affected by the hypercholesterolemic condition of ApoE animals or by ovariectomy.

## Discussion

One of the hallmarks of atherosclerosis in humans is the impairment of endothelial function [5]. The ApoE-deficient mouse is considered one of the most relevant models for atherosclerosis because this animal develops spontaneous arterial lesions [2,3]. Resistance vessels of male ApoE mice generally present normal endothelial function on a normal chow diet [12,13] and endothelial dysfunction is only observed in high-fat diet conditions [25]. In contrast, female ApoE mice, even on a normal diet, show endothelial dysfunction in aorta and cerebral resistance vessels [9,14]. In the present study, we show that the isolated mesenteric arteriolar bed of female ApoE-deficient mice fed a normal diet also presents endothelial dysfunction, as indicated by the marked decrease in ACh-induced vasorelaxation. The impairment of vasorelaxation does not appear to involve the vascular smooth muscle because the endothelium-independent vasorelaxation to SNP was preserved in the female ApoE-deficient mouse. On the other hand, in the present study we observed a normal NE-induced vasoconstriction in resistance vessels of female ApoE-deficient mice, corroborating the finding by others showing preserved responses to NE in aorta rings of female ApoE-deficient mice under both normal and high-fat diets [8]. In this model, the normal vascular responsiveness to NE seems to be related only to females because in previous study it was found an exacerbated NE-induced vasoconstriction in male mice [12].

Gender is a potent risk factor for cardiovascular diseases [26]. In contrast with females, males have an increased prevalence of atherosclerotic-based diseases, such ischemic heart disease, stroke and aortic aneurysms [27,28]. In the present study, we investigated resistance vessel responsiveness in both normal and spontaneously atherosclerotic female mice. In agreement with others [29-31], mice subjected to the removal of ovaries presented a smaller uterus weight in comparison with noncastrated mice. In the present study, we observed that ovariectomy led to a significant reduction in ACh-induced vasorelaxation in female C57 mice. Although impairment of endothelial function has been observed in conductance vessels of castrated female mice, dysfunction of resistance vessels of C57 animals had not yet been reported. Based on other studies, this finding could be explained by the fact that estrogen has direct beneficial effects on endothelial function by augmenting prostacyclin and NO synthesis [17,32-34].

Contrasting with males [12,13], we observed that female ApoE-deficient mice on a normal chow diet show endothelial dysfunction of resistance vessels. Similar results have been observed by others in the aorta and cerebral arterioles of female ApoE-deficient mice [9,14]. These observations corroborate the finding that female ApoE-deficient mice present a higher vascular lesion area than male mice [35], which appears to be related to direct effects of estrogen on the specific immune activation on female mice. In the present study, the ovariectomy of female ApoE-deficient mice did not cause additional damage to the endothelial function of resistance vessels, probably because the animals already presented marked endothelial dysfunction. This finding seems to be related only to the ApoE-deficient mouse because it is well known that in other models of cardiovascular diseases estrogen deficiency leads to endothelial dysfunction [17,22]. Similar to C57 female mice, we did not observe any effect of ovariectomy on the NE-induced vasoconstriction in resistance vessels of female ApoE-deficient mice, corroborating the finding that female 17β-estrogen receptor knockout mice did not present any change in NE-induced vasoconstriction [36].

In conclusion, the current findings demonstrated an impairment of endothelial function in resistance vessels of spontaneously atherosclerotic (ApoE-deficient) female mice compared with normal (C57) female mice. The endothelial dysfunction was so marked that ovariectomy, which impaired the endothelial function in C57, did not cause additional damage in ApoE-deficient mice. Further studies will be required to elucidate the mechanisms involved in the endothelial dysfunction in these animals. We speculate that increased superoxide anion production and reduced activity of endothelial nitric oxide synthase could contribute to the endothelial dysfunction in female ApoE-deficient mice.

## Competing interests

The authors declare that they have no competing interests.

## Authors' contributions

MSC carried out the animal experiments, analysis of the data, statistics and drafted the manuscript. ALG carried out the ovariectomy surgery and participated in manuscript preparation. SSM participated in the design and co-supervision of the study and in the critical revision of the manuscript. ECV conceived the study, participated in its design and supervision and in the critical revision of the manuscript. All authors read and approved the final manuscript.
